# Therapy-related myeloid neoplasms following treatment for multiple myeloma—a single center analysis

**DOI:** 10.1007/s00277-022-04775-1

**Published:** 2022-03-09

**Authors:** A. Boquoi, S. M. Banahan, A. Mohring, I. Savickaite, J. Strapatsas, B. Hildebrandt, G. Kobbe, N. Gattermann, R. Haas, T. Schroeder, U. Germing, R. Fenk

**Affiliations:** 1 Department of Hematology, Oncology and Clinical Immunology, University Hospital Duesseldorf, Heinrich-Heine-University, Duesseldorf, Germany; 2 Institute of Human Genetics, Heinrich-Heine-University, Duesseldorf, Germany; 3 Department of Hematology and Stem Cell Transplantation, University Medicine Essen, Essen, Germany

**Keywords:** MDS, AML, Multiple myeloma, Therapy-related, tMDS

## Abstract

Myelodysplastic syndromes (MDS) and acute myeloid leukemia (AML) can be late complications following mutagenic treatment. Limited data is available on the outcome of patients developing therapy-related MDS and AML after treatment for multiple myeloma (MM). We identified 250 patients with therapy-associated MDS or AML in the Duesseldorf MDS registry. Of those, 50 patients were previously diagnosed with multiple myeloma (mm-MDS/AML). We compared them to patients with de novo MDS (*n* = 4862) and to patients with MDS following other underlying diseases (tMDS) (*n* = 200). mm-MDS patients and tMDS patients showed similar karyotypes and degrees of cytopenia. However, mm-MDS patients had significantly higher blast counts and more often belonged to the high-risk group according to the International Prognostic Scoring System (IPSS) (both *p* < 0.05). Although the rate of progression to AML was similar in mm-MDS and tMDS, both transformed significantly more often than de novo MDS (*p* < 0.05). Median overall survival of patients with mm-MDS (13 months; range: 1–99) and tMDS (13 months; range 0–160) was also similar yet significantly shorter than patients with de novo MDS (32 months; range 0–345 months; p < 0.05). Furthermore, survival of mm-MDS patients was not affected by myeloma activity. Despite significantly more high-risk disease and higher blast cell counts, myeloma-associated MDS-patients show features akin to other tMDS. Survival is similar to other tMDS and irrespective of myeloma remission status or transformation to AML. Thus, patient outcome is not determined by competing clones but rather by MDS governing the stem cell niche.

**Supplementary Information:**

The online version contains supplementary material available at 10.1007/s00277-022-04775-1.

## Introduction

Myelodysplastic syndromes (MDS) and acute myeloid leukemia (AML) can represent late complications following cytotoxic treatment and are summarized as the distinct category “therapy-related myeloid neoplasms” in the current World Health Organization (WHO) classification [1]. They account for approximately 10–20% of all cases of MDS and AML, and their incidence is likely to rise given the increasing number of cancer survivors [2]. Most patients who develop therapy-related myeloid neoplasms previously received alkylating agents, topoisomerase II inhibitors, antimetabolites, and/or irradiation [3]. Some therapy-related myeloid neoplasms also occurred after intensive immunosuppressive treatment or radioiodine treatment [4, 5].

The prognosis of tMDS is poor with a life expectancy of typically less than a year [6]. Up to 90% of patients have high risk clonal karyotypes [7, 8]. Progression to AML is usually rapid and often accompanied by relative resistance to conventional chemotherapy [9].

In this retrospective study, we analyzed clinical characteristics, cytogenetic data, risk of AML transformation, and probability of survival of 50 patients with MDS/AML following multiple myeloma therapy (mm-MDS). We also compared them with therapy-associated MDS/AML due to other underlying diseases (tAML/tMDS) and with de novo MDS. Furthermore, we assessed whether myeloma activity affected the outcome after MDS/AML diagnosis.

## Material and methods

### Patients

Between 1968 and 2011, 5362 patients with MDS were entered into the Düsseldorf MDS Registry. Of those, 250 patients were classified as tMDS/tAML. Fifty of these patients had previously been diagnosed with MM (Supp. Fig. S1a) [10, 11].

In patients with prior MM, patient characteristics at MM diagnosis, the type of anti-myeloma treatment, and the response to treatment were recorded. In case of mm-MDS progressing to a more advanced type of MDS or AML, time to progression and time to overt AML were calculated from the date of initial diagnosis of the therapy-related myeloid neoplasm. Patient characteristics including WHO 2016 MDS type, IPSS, age, date of diagnosis, survival time, and blood counts were available in the Duesseldorf MDS Registry.

Karyotypes were reported in accordance with the International System for Human Cytogenetic Nomenclature and graded according to the International Prognostic Scoring System (IPSS), while cytogenetic data was reported following the ELN guidelines [12].

All patients gave written informed consent to participate in the registry. The study was approved by the ethics committee of the Medical Faculty of Heinrich Heine University Duesseldorf (registration numbers 3973 und 3008).

### Statistics

Median and ranges were calculated to describe patients’ characteristics. Overall survival and time to AML evolution were estimated according to the Kaplan–Meier method. The log-rank test was used for comparison of overall survival between subgroups, whereas cross-tabulation and the χ2 test or Fisher’s exact test were employed for comparison of biological variables. Medians were compared with the non-parametric Mann–Whitney Test. Statistical analyses were carried out using Excel (Microsoft) and SPSS for Windows (SPSS Inc. Chicago, IL, USA). *p*-levels < 0.05 were considered significant.

## Results

### Patients characteristics

We identified 250 therapy-associated myeloid neoplasms in our cohort. One hundred seventeen patients (46.8%) had previously suffered from hematological malignancies, 104 patients (41.6%) had been treated for solid tumors, and 17 (6.8%) had been diagnosed with other diseases. Sufficient data was missing for 12 patients (4.8%). Of the 117 patients with hematologic malignancies, 50 patients (17 female, 34 male) had received treatment for multiple myeloma (43%). Other previous hematologic diseases included non-Hodgkin lymphoma (36%), Hodgkin lymphoma (13%), acute myeloid leukemia (6%), acute lymphoblastic leukemia (1%), and Waldenström’s macroglobulinemia (1%) (Supp. Fig. S1b).

### Patient characteristics of mm-MDS patients

The median age at MM diagnosis was 61 years (range 26–80 years). The median age at mm-MDS/AML diagnosis was 68 years (range 33–85 years). The median time between MM diagnosis and the onset of MDS was 5.5 years (range 0–28.5 years).

Of patients with myeloma-associated MDS/AML, 84.4% had received conventional chemotherapy, mostly anthracyclines and alkylating agents. Of those, 94% had received melphalan, 30% as high-dose conditioning for autologous stem cell transplantation. Of these, 73% had received a single transplant and 27% a double transplant. The remaining 70% of patients had received oral melphalan with 8 mg/m^2^ plus oral prednisone 60 mg both over 4 days of a 28-day cycle according to standard protocol [13]. The median of cycles received was 24 (range 1–50). Sixteen percent had received novel agents including immunomodulatory drugs and proteasome inhibitors. Outcome showed no difference between the previous treatment received, so we grouped them together.

According to the WHO classification of 2016, 7 of the 50 patients with mm-MDS presented with single-lineage dysplasia (MDS-SLD), 10 patients with multi-lineage dysplasia (MDS-MLD), 1 patient with single-lineage dysplasia and ring sideroblasts (MDS-RS-SLD), 13 patients with multi-lineage dysplasia and ring sideroblasts (MDS-RS-MLD), 7 patients with MDS with excess blasts I (MDS-EB1), and 8 patients with excess blasts II (MDS-EB2). In addition, there were 2 patients with CMML-1 and 1 patient with CMML-2. One patient showed isolated del(5q).

Cytogenetic data was available for 48 patients with mm-MDS at the time of MDS diagnosis: 68% had an abnormal karyotype, and 58% had a complex karyotype. The chromosomes most frequently affected were chromosomes 5 (32%), 7 (25%), 17 (18%), 20 (18%), and 21 (18%).

Based on IPSS prognostic risk score, no mm-MDS patient was stratified as low-risk, 24 patients as intermediate-risk I (57%), 9 patients as intermediate-risk II (21%), and 9 patients as high-risk (21%) (data was available for 42 patients).

Based on IPSS-R prognostic risk score, 2 patients were stratified as very low risk (7%), 8 patients as low (28%), 5 patients as intermediate (18%), 4 patients as high (15%), and 9 patients as very high (32%) (data was available for 28 patients).

Mutation analyses were not performed routinely and were not available for these patients.

### mm-MDS/mm-AML treatment

Data on the treatment of tMDS/tAML was available for 37 (74%) of the 50 patients. Following the diagnosis of tMDS/tAML, 17 patients (47%) received best supportive care only (including transfusions, iron chelation, and hematopoietic growth factors) or low dose chemotherapy (low-dose cytarabine and/or hydroxyurea) or valproate with or without all-*trans* retinoic acid.

Intensive induction chemotherapy using various cytarabine/anthracycline-based regimens was employed in 6 patients (16%), while upfront allogeneic hematopoietic stem cell transplantation was performed in 6 patients (16%). Five patients (14%) received epigenetic treatment with a DNA methyltransferase inhibitor (5-azacitidine). Three patients (8%) deceased without having received any therapy.

### Comparison between myeloma-therapy related MDS, other therapy-related MDS and de-novo MDS

Table 1 and Supp Fig. S1 show detailed comparison between the three groups. mm-MDS patients were significantly younger at diagnosis compared to de-novo MDS patients (*p* < 0.05). However, they were significantly older than all other therapy-associated MDS patients (*p* < 0.05).

**Table 1:** patient characteristics and comparison between cohorts

Patient characteristics	mm-MDS	t-MDS		de novo MDS	mm-MDS versus t-MDS	mm-MDS versus de novo MDS	t-MDS versus de novo MDS
	(*n* = 50)	(*n* = 200)		(*n* = 5112)	*p*-value	*p*-value	*p*-value
**Age at MDS diagnosis**	67.8	64.3		71.9			
(median years, range)	(32.5–84.6)	(21.2–85.4)		(18.3–105.2)	0.1579	0.0073	< 0.0001
Gender							
(%) (m: f)	67:33	48: 52		57: 43	0.0268	0.2324	0.0201
n (m: f)	33:17	96:104		2876:2236			
**Hematologic parameters at MDS diagnosis**							
Hemoglobin (g/dl)	8.4	9		9.5	0.1159	0.0012	0.0049
(median, range)	(5.2 – 14.9)	(1.9 – 15.1)		(2.2 – 17.5)			
Leukocytes (*10^3^/µl)	24	30		41	0.08	< 0.0001	< 0.0001
(median, range)	(5 – 76)	(3 – 980)		(0.04 – 1500)			
Thrombocytes (*10^3^/µl)	68	75		123	0.7037	0.0002	< 0.0001
(median, range)	(3 – 588)	(6 – 444)		(1 – 371)			
Granulocytes (/µl)	1369	1443		2088	0.3593	0.0006	< 0.0001
(median, range)	(46 – 7176)	(49 – 35,280)		(0.6 – 91,636)			
**Type of cytopenia at MDS diagnosis (%)**							
Anemia	18	10		36			
Leukopenia	3	2		1			
Thrombopenia	0	3		2			
Anemia & Leukopenia	18	6		14			
Anemia & Thrombopenia	10	26		15			
Leukopenia & Thrombopenia	0	5		3			
Pancytopenia	50	48		26			
Normal	3	1		2			
Mono-/Bicytopenia vs. Pancytopenia					0.8591	0.0014	< 0.0001
Bicytopenia vs. Pancytopenia					0.1337	0.0283	0.0070
**IPSS Score (%)**							
Low	0	11	26				
Int-1	58	41	35				
Int-2	21	32	17				
High	21	16	22				
High vs. low, Int-1, Int-2					0.4781	> 0.9999	0.1567
Low vs Int-1, Int-2, High					0.0371	< 0.0001	0.0002
**Criteria IPSS-Score**							
**IPSS Blasts (%)**							
0–4	55	52	60				
5–10	21	23	20				
11–20	12	21	14				
21–29	12	4	6				
< 10% vs. > 10% Blasts					1	0.7765	0.1992
< 20% vs. > 20% Blasts					0.0476	0.2345	0.2293
**IPSS Cytopenia (%)**							
0–1	24	32	56				
2–3	76	69	44				
0/1 Cytopenia vs. 2/3 Cytopenia					0.4489	0.0001	< 0.0001
**IPSS Cytogenetics (%)**							
Low	38	42	65				
Intermediate	24	11	17				
High	38	47	18				
High vs. Non-high					0.4162	0.0125	< 0.0001
Low vs. Non-low					0.8359	0.0031	< 0.0001

Gender distribution also showed significant differences: both mm-MDS and de novo MDS affected significantly more males than females (*p* < 0.05), while other therapy-related MDS patients showed a balanced male to female ratio.

mm-MDS showed significantly more blasts in the bone marrow than other tMDS and de novo MDS (*p* < 0.05). Also, we found significantly less mm-MDS patients in the IPSS low-risk category compared to tMDS or de novo MDS patients (*p* < 0.05).

Complete blood counts were similar in mm-MDS and tMDS without significant differences (*p* > 0.05). However, both mm-MDS and tMDS showed significant differences compared to de novo MDS (*p* < 0.05). While median hemoglobin values were lowest in mm-MDS (8.4 g/dl, range 5.2–14.9), with a trend to be worse than in tMDS (9.0 g/dl, range 1.9–15.1; *p* > 0.05), median leukocyte and platelet counts were significantly lower in both mm-MDS and tMDS patients than de novo MDS patients (*p* < 0.05).

Degrees of cytopenia as well as lineages affected were similar in mm-MDS and tMDS without significant differences (*p* > 0.05). However, both mm-MDS and tMDS showed significant differences compared to de novo MDS with both displaying more single and double lineage cytopenia and pancytopenia (p < 0.05).

Karyotype anomalies were also similar in mm-MDS and tMDS patients (*p* > 0.05), However, both showed significantly worse karyotype anomalies when compared to de novo MDS. When we grouped patients in low versus non-low and high versus non-high, we found statistical differences for both mm-MDS and tMDS versus de novo MDS (*p* < 0.05).

### Survival

**Fig. 1:**
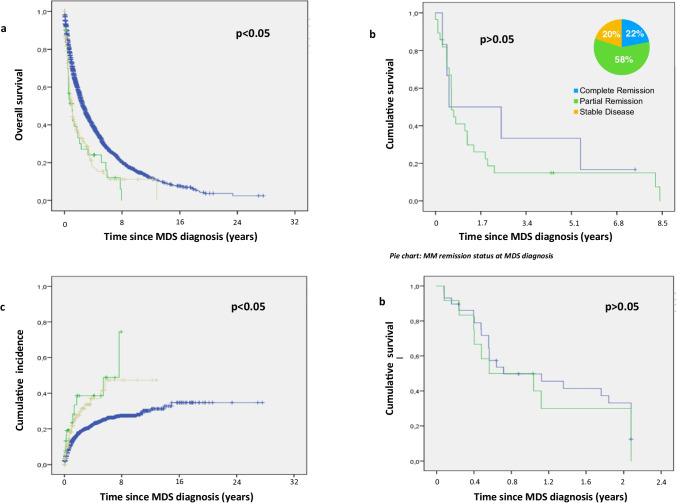
**a** median survival in mm-MDS (green), tMDS (brown) and de novo MDS (blue). **b** mm-MDS survival of pts in complete remission (blue) and in partial remission (green). Pie chart: MM remission status at MDS diagnosis. **c** Cumulative Incidence of AML in mm-MDS pts (green), tMDS (brown), and de novo MDS (blue). **d** Survival over time since MDS diagnosis in mm-MDS with AML transformation (green) and without AML transformation (blue)

Median overall survival of both mm-MDS and all other tMDS was similar with 13 months each (mm-MDS range 0–99 months; tMDS range 0–160 months). Median survival of all de novo MDS patients was significantly longer with 32 months after diagnosis (range 0–345 months, *p* < 0.05 for both) (Fig. 1a).

### Progression and transformation to advanced MDS or AML

Of all 50 mm-MDS patients, 6 patients progressed to an advanced type of MDS (12%) (MDS-RS to MDS-EB2 in 1 patient, MDS-MLD to CMML-1 in 1 patient, del(5q) to CMML-1 in 1 patient, CMML-1 to CMML-2 in 1 patient, MDS-RS to MDS-EB2 in 1 patient, MDS-EB1 to MDS-EB2 in 1 patient). Later, 3 of these 6 patients showed disease progression to AML. Median time to progression was 12 months (range 9–21 months).

Of all 50 mm-MDS patients, 13 developed AML (26%). The median age at mm-AML diagnosis was 70 years (range 55–80 years). Progression to AML occurred most often in MDS types MDS-MLD and MDS-EB1 (each 23%), while 15% of patients with MDS MDS-EB2 progressed to AML. Median time to progression from MDS to AML was 5 months (range 0.5–68 months). The majority of patients developed AML M2 (60%), 20% developed M1, and 20% M6.

Cytogenetic data was available for 8 mm-AML patients. Applying the recently proposed risk stratification of the European Leukemia Network [14], no patient belonged to the favorable genetic risk group (0%), 2 patients to the intermediate group (25%), and 6 patients to the adverse risk group (75%). Mutation analyses were not performed routinely and were not available for these patients.

Transformation to AML was similar in mm-MDS and tMDS (24% of mm-MDS and 19% of tMDS 12 months after MDS diagnosis and 39% of mm-MDS and 34% of tMDS at 36 months, *p* > 0.05). However, AML transformation occurred significantly more often in mm-MDS and tMDS patients than in de novo MDS patients (*p* < 0.05, Fig. 1c).

Progression to AML had no significant effect on survival, though, which was similarly poor in mm-MDS with or without AML transformation (7 months versus 11 months, *p* > 0.05, Fig. 1d).

### Impact of myeloma activity on prognosis

To analyze the prognostic impact of the underlying disease on the outcome of mm-MDS, we recorded myeloma activity according to IMWG response criteria at the time of mm-MDS diagnosis. mm-MDS patients in complete remission showed a median survival of 6 months (range 0–35 months) and patients with residual myeloma activity (partial remission, non-responders) lived a median of 7 months (range 6–8 months, *p* > 0.05) (Fig. 1b).

## Discussion

The field of multiple myeloma (MM) has seen major therapeutic progress over the last 15 years.

Bortezomib-based induction therapy and lenalidomide maintenance after high-dose melphalan plus autologous stem cell transplantation have become standard of care for eligible MM patients [15]. Concerns about second primary malignancies (SPM) were raised, though, when an increased cumulative incidence of hematological SPM (mainly AML and MDS) in lenalidomide-treated patients was reported [16, 17]. However, multivariate analysis suggested that the risk of hematological SPM may be significantly driven by prior or concurrent use of melphalan and due to longer overall survival [18, 19].

Our analysis shows that the majority of mm-MDS patients present with high-risk MDS or AML, as reflected by blast count (12% showing more than 20% blasts), karyotype (38% poor risk), IPSS (no patients with low risk disease), or rapid disease progression (24 and 39% transformation of MDS to AML, respectively).

In terms of karyotype, this is comparable to data from large series of patients with tMDS or tAML, which showed an abnormal karyotype in 75% and 92% of all cases, while an abnormal karyotype was observed in only 51% and 52% of patients with de novo AML/MDS, respectively [20, 21].

We observed chromosomes 5, 7, 17, and 22 to be most frequently affected in our series. Myeloma patients, however, most commonly display numerical abnormalities with gains of chromosomes 15, 9, and 3 only followed by chromosomes 19, 11, 7, 21, and 5 [22, 23]. Smith et al. found clonal abnormalities of chromosomes 5 and 7 to be most common among tMDS patients [24]. More recently, detailed pathological analysis of myeloid neoplasms secondary to MM yielded evidence of complex cytogenetic abnormalities or unbalanced aberrations mostly of chromosomes 5 and 7, further supporting our data [25].

Increased SPMs in myeloma patients have been associated with older age [26, 27]. Our data confirms mm-MDS patients to be younger than de novo MDS patients but older than tMDS patients. Previous therapy, especially the extensive use of alkylators in the younger age group, predisposes MM patients to develop MDS earlier than individuals who develop de novo MDS. However, mm-MDS patients are still older than other tMDS patients because the age at MM diagnosis is generally higher than the age at diagnosis of other cancer types that require chemotherapy. Especially women with breast cancer might affect this age difference with a median age of 62, while the median age for MM diagnosis is 69 years [28, 29].

Similarly, male sex has often been associated with increased SPMs in MM [30]. Mahindra et al. reported that women with MM had a significantly lower risk of new cancer compared with men [31]. Our results confirm this gender distribution in MM and also corroborate the different picture observed in other tMDS, where women who previously received breast cancer therapy contribute a large share to the tMDS patient population.

Several authors have shown that overall survival of patients with tMDS/AML is shorter than that of patients with de novo MDS, irrespective of the treatment applied [32, 33]. We also observed a statistically significant difference in overall survival between patients with mm-MDS and patients with de novo MDS. However, there was no difference in survival between mm-MDS and other tMDS despite a more aggressive phenotype with more pronounced cytopenias and higher blast cell counts in mm-MDS. Similarly, we observed mm-MDS patients to transform to AML significantly more often than tMDS or de novo MDS, but again this was not reflected by a difference in the already poor survival.

Previous studies showed that symptomatic MM leads to functional impairment and mitigation of hematopoietic stem cells suggesting active MM to further exacerbate secondary MDS [34]. However, survival of patients in our cohort was not affected by myeloma activity. By using multidimensional flow, several authors saw MDS-associated phenotypic abnormalities already at MM diagnosis: Matarraz et al. found myelodysplasia-associated immunophenotypic alterations in approximately 47% of patients with symptomatic MM and 33% of patients with smoldering MM [35]. Importantly, these immunophenotypic alterations correlated with genetic/morphologic evidence of clonal hematopoiesis in myeloid lineage cells and infrequently re-emerged after stem cell transplant suggesting a significant role of non-treatment-related factors [36, 37]. Similar to our results, these patients showed significantly higher age and experienced more frequently hematological toxicity including anemia during treatment [38].

In the era of novel therapies, melphalan may seem less and less relevant. However, Gay et al. recently noted in the FORTE trial that patients receiving melphalan conditioning plus autologous transplant showed improved progression-free survival when compared to patients treated without melphalan conditioning plus transplant [39]. Furthermore, Mateos et al. recently published the ALCYONE trial which lead to the approval of daratumumab, bortezomib, prednisone, and oral melphalan for non-transplant eligible MM patients [40]. Thus, melphalan is and will continue to be widely used demonstrating the relevance of our data.

To avoid bias due to different therapies previously received, we performed separate analyses and found no significant difference between the cohorts. Thus, we grouped them together. A similar cohort was analyzed by Pemmaraju N et al. in which 68% of mm-MDS patients had received conventional therapy, 42.6% high dose Melphalan and ASCT, and 14.9% novel agents [41]. The outcome was also irrespective of specific MM treatment.

## Conclusion

Despite significantly more high-risk disease, higher blast cell counts, and more frequent progression to AML, myeloma-associated MDS-patients show features akin to other tMDS. Survival is similar to other tMDS and irrespective of myeloma activity or transformation to AML. Thus, patient outcome is not determined by formally crossing the line from MM to MDS/AML but rather by MDS governing the stem cell niche. Since multidimensional flow can reveal MDS-associated phenotypic alterations already at MM diagnosis and melphalan remains a crucial element of current myeloma therapy and therefore risk factor to develop MDS, more effort is needed to early identify patients at risk of developing MDS/AML.

## Supplementary Information

Below is the link to the electronic supplementary material.

Supplementary file1 (PDF 401 KB)
