# Supplementary material for: Therapy-related myeloid neoplasms following treatment for multiple myeloma—a single center analysis
Source: Ann Hematol. 2022 Mar 9;101(5):1031–8. doi: 10.1007/s00277-022-04775-1 (PMC8993729; doi:10.1007/s00277-022-04775-1)
Supplement: Supplementary file 1 — Supplementary file1 (PDF 401 KB) [file 277_2022_4775_MOESM1_ESM.pdf]

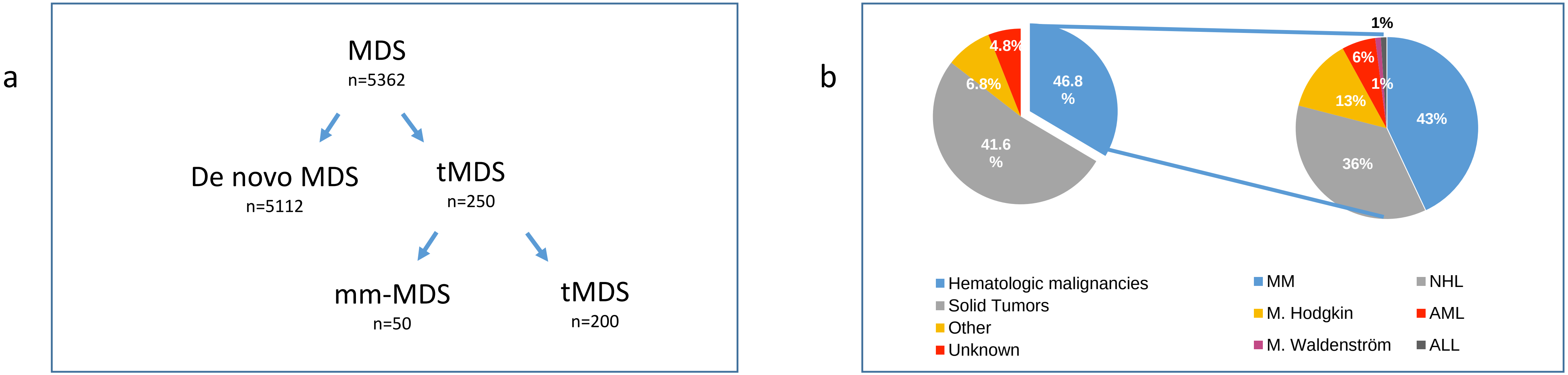

c

	De novo MDS	versus		mm-MDS	versus		tMDS
Age		>	p<0.05		>	p<0.05	
IPSS low		>	p<0.05		<	p<0.05	
Blast count		<	p<0.05		>	p>0.05	
Complete Blood Count		>	p<0.05		=	p>0.05	
Lineages affected		<	p<0.05		=	p>0.05	
Degrees of cytopenia		<	p<0.05		=	p>0.05	
Poor Karyotype		<	p<0.05		=	p>0.05	

Figure 1: a) overview of patient recruitment b) overview of previous malignancies c) comparison between mm-MDS, other tMDS and de novo MDS
